# GATA3 and TRPS1 are distinct biomarkers and prognostic factors in breast cancer: database mining for GATA family members in malignancies

**DOI:** 10.18632/oncotarget.16160

**Published:** 2017-03-13

**Authors:** Hao-Yu Lin, De Zeng, Yuan-Ke Liang, Xiao-Long Wei, Chun-Fa Chen

**Affiliations:** ^1^ Department of Breast and Thyroid Surgery, The First Affiliated Hospital of Shantou University Medical College, Shantou, China; ^2^ Department of Breast Medical Oncology, Cancer Hospital of Shantou University Medical College, Shantou, China; ^3^ ChangJiang Scholar's Laboratory of Shantou University Medical College, Shantou, China; ^4^ Department of Medical Oncology, University of Groningen, University Medical Center Groningen, Groningen, The Netherlands; ^5^ Department of Pathology, Cancer Hospital of SUMC, Shantou, China

**Keywords:** GATA, breast cancer, database mining, prognostic values, chemosensitivity

## Abstract

GATA transcription factors are zinc finger DNA binding proteins that activate transcription during development and cell differentiation. To date, 7 members of GATA family have been reported. However, the expression patterns and the exact roles of distinct GATA family members contributing to tumorigenesis and progression of breast cancer (BC) remain to be elucidated. Here, we studied the expression of GATA transcripts in a variety of tumor types compared with the normal controls using the *ONCOMINE* and *GOBO* databases, along with their corresponding expression profiles in an array of cancer cell lines through *CCLE* analysis. Based on Kaplan-Meier plotter, we further investigated the prognostic values of GATA members specifically high expressed in BC patients. It was found that, when compared with normal tissues, GATA3 and TRPS1 were distinctly high expressed in BC patients among all GATA members. GATA3 expression was significantly associated with ESR1, while TRPS1 was correlated with ERBB2. In survival analysis, GATA3 and TRPS1 mRNA high expressions were correlated to better survival in BC patients, and TRPS1 high expression was significantly associated with longer RFS in patients who have received chemotherapy. These results suggest that GATA3 and TRPS1 are distinct biomarkers and essential prognostic factors for breast cancer.

## INTRODUCTION

Breast cancer (BC) remains the top cancer and the principle cause of death from cancer in women worldwide [1]. Disorder in development of mammary epithelia is a prominent process contributing to tumorigenesis of BC [2].

Abnormality in a variety of genes, as well as signaling pathways, is reported to be responsible for aberrant growth and differentiation of mammary epithelia, such as GATA, Notch and Wnt, etc [3–5]. GATA has been identified as one of the transcription factors that play an essential role during epithelial proliferation [6–8], which comprised family members sharing highly conserved zinc fingers that recognize the motif WGATAR to mediate DNA binding and protein interactions [9].

GATA1, GATA2 and GATA3 were termed hematopoietic GATA factors [10, 11], while GATA4, GATA5, and GATA6 were categorized as endodermal GATA factors [12]. GATA1 and GATA2 play pivotal roles in regulating cell cycle or proliferation [13]. GATA3 is estimated to be the most abundant transcription factor in luminal epithelial cells expression and is required for normal development of the mammary gland [14, 15]. GATA4, GATA5 and GATA6 are expressed in distinct but overlapping patterns [16–18]. GATA4 and GATA5 tend to mark fully differentiated epithelial cells, while GATA6 expresses in the immature proliferating cells [19]. TRPS-1 (trichorhinophalangeal syndrome-1) is a novel GATA transcription factor that has been found to be a critical activator of mesenchymal-to-epithelial transition (MET) during embryonic development in a number of tissues, including bone, cartilage and kidney [20].

Recently, Marjokein and Zheng et al. have reviewed the emerging role of GATA transcription factors in malignancies [6, 9], suggesting the distinctive role of individual GATA member in cancer development and progression. Although GATA has been identified as a crucial transcription factors in a variety of hematogenous malignancies and solid tumors [9, 21], the functions of different GATA members in contribution to tumorigenesis in BC are largely unknown.

In the current study, we extended the research field to breast cancers based on large databases, with purpose of determining the expression pattern of distinct GATA family members in breast cancer *VS*. normal tissues and the correlations with characteristic molecular markers, as well as their corresponding prognostic values in breast cancer.

## RESULTS

### GATA3 and TRPS1 are distinctively overexpressed in breast cancer among all GATA family members

**Figure 1:**
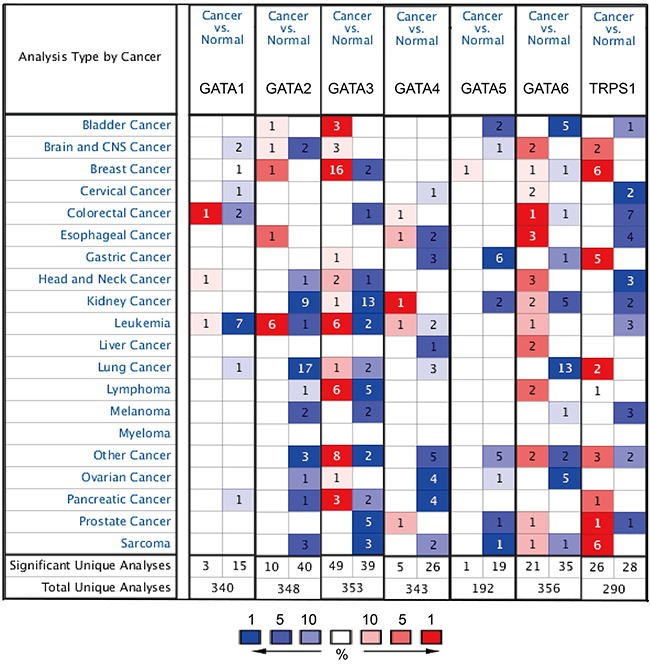
The mRNA expression pattern of GATA family members in different tumor types This graphic showed the numbers of datasets with statistically significant mRNA over-expression (red) or down-expression (blue) of the target gene (cancer vs. normal tissue). The *p* value threshold is 0.01. The number in each cell represents the number of analyses that meet the threshold within those analysis and cancer types. The gene rank was analyzed by percentile of target gene in the top of all genes measured in each research. Cell color is determined by the best gene rank percentile for the analyses within the cell.

**Figure 2:**
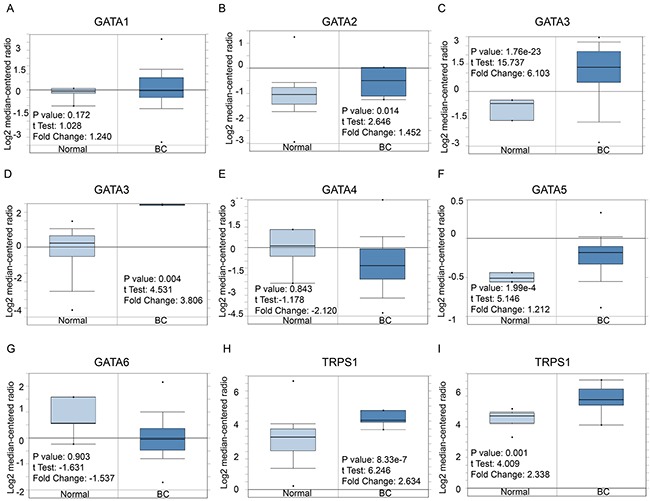
GATA family analysis in Breast cancer (*ONCOMINE* database) Box plots derived from gene expression data in *ONCOMINE* comparing expression of a specific GATA family member in normal and BC tissue. The *p* value was set up at 0.01 and fold change was defined as 2. **(A)** Comparison of GATA1 mRNA expression. **(B)** Comparison of GATA2 mRNA expression. **(C-D)** Comparison of GATA3 mRNA expression. **(E)** Comparison of GATA4 mRNA expression. **(F)** Comparison of GATA5 mRNA expression. **(G)** Comparison of GATA6 mRNA expression. **(H)** Comparison of TRPS1 mRNA expression.

Hitherto, 7 GATA family members have been identified in human cancers, including hematological malignancies and solid tumors (Figure 1). *ONCOMINE* analysis revealed that GATA3 mRNA expression was significantly higher in BC than normal samples across a wide variety of datasets in different cancer types. GATA3 transcripts were 6.103 fold elevated in breast cancer samples as compared with normal tissues in a dataset with 593 samples that derived from TCGA (the Cancer Genome Atlas) database (Figure 2C). In another dataset from Zhao's study [22], GATA3 was 3.806 fold elevated in breast cancer samples as compared with normal tissues (*p=*0.004) (Figure 2D).

Moreover, the analysis also demonstrated that the transcription level of TRPS1 was significantly elevated in breast cancer versus. normal samples. TRPS1 was 2.634 fold increased (*p=*8.33e-7) in the largest dataset with 593 samples from TCGA. Consistently, in another dataset from Sorlie's study [23], TRPS1 was 2.338 fold increased in cancer *VS*. normal samples (*p=*0.001).

However, no significant difference was found in the mRNA level of other GATA members, including GATA1 (1.24 fold change, *p*=0.172), GATA2 (1.452 fold change, *p*=0.014), GATA4 (2.12 fold change, *p*=0.843), GATA5 (1.212 fold change, *p*=1.99e-4) and GATA6 (1.537 fold change, *p*=0.903), between breast cancer samples and normal controls (Figure 2A, 2B, 2E, 2F and 2G).

**Figure 3:**
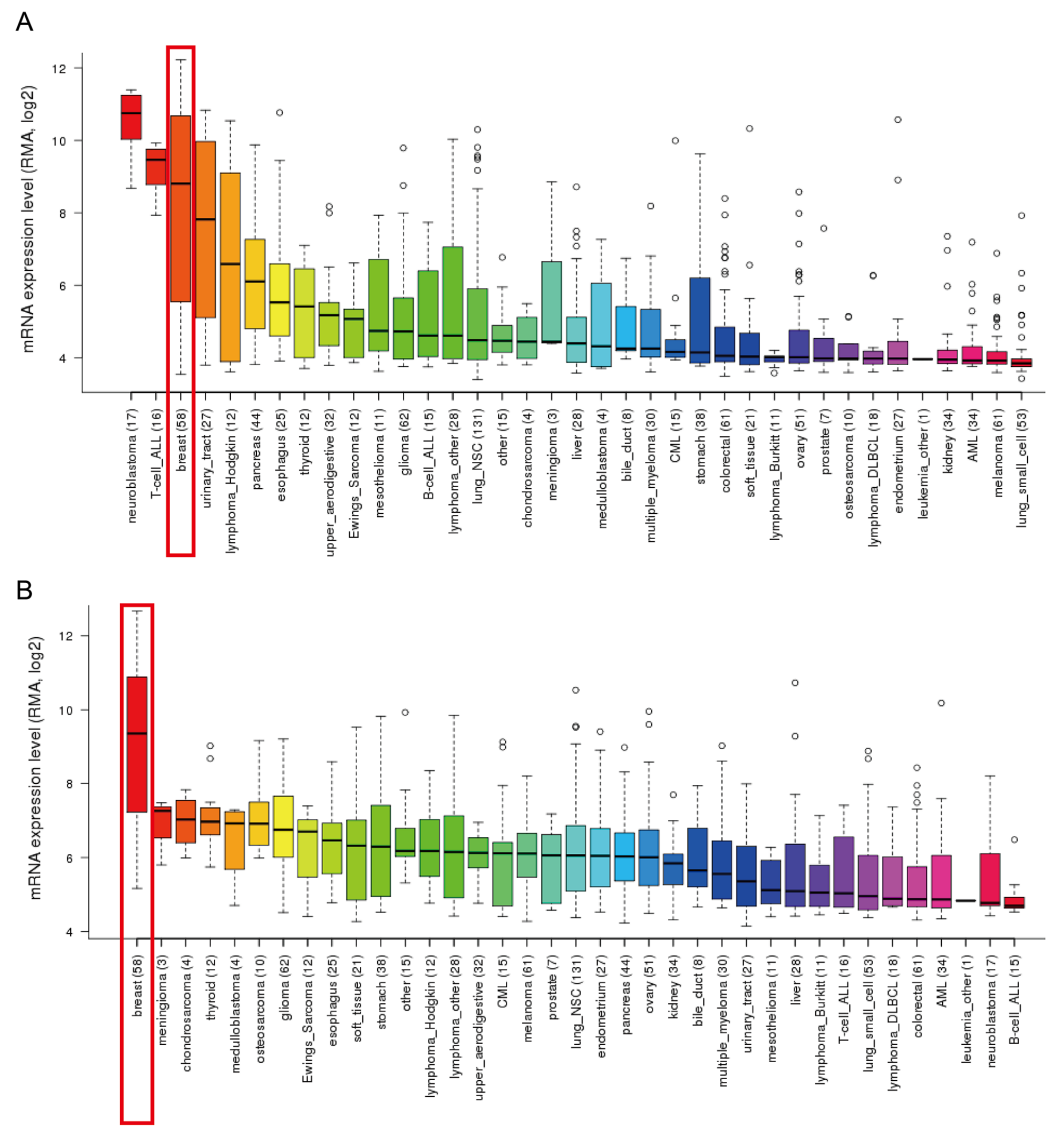
GATA3 and TRPS1 were distinctively high expressed in breast cancer cell lines from *CCLE* analysis **(A)** The mRNA expression level of GATA3 ranked the third highest in a variety of cancer cell line, behind that of retinoblastoma and T-cell-ALL, (shown in red frame). **(B)** The mRNA expression level of TRPS1 ranked the highest in breast cancer among different cancer cell types, (shown in red frame).

In addition, *CCLE* analysis was consistent with that of *ONCOMINE* demonstrating that GATA3 and TRPS1 were distinctively up-regulated in breast cancer cell lines, while other GATA members were present at a low transcription level or absent in breast cancer cells (Figure 3A and 3B). These results implied that GATA3 and TRPS1 were different from other GATA members that distinctively high expressed in breast cancer, suggesting they might play unique roles in the development of breast cancer. The mRNA expressions of other GATA members evaluated in different breast cancer cell lines were provided in Supplementary Figures (Supplementary Figures 1-5).

### The co-expression analysis of GATA3 and TRPS1 in different molecular subtypes of breast cancer

**Figure 4:**
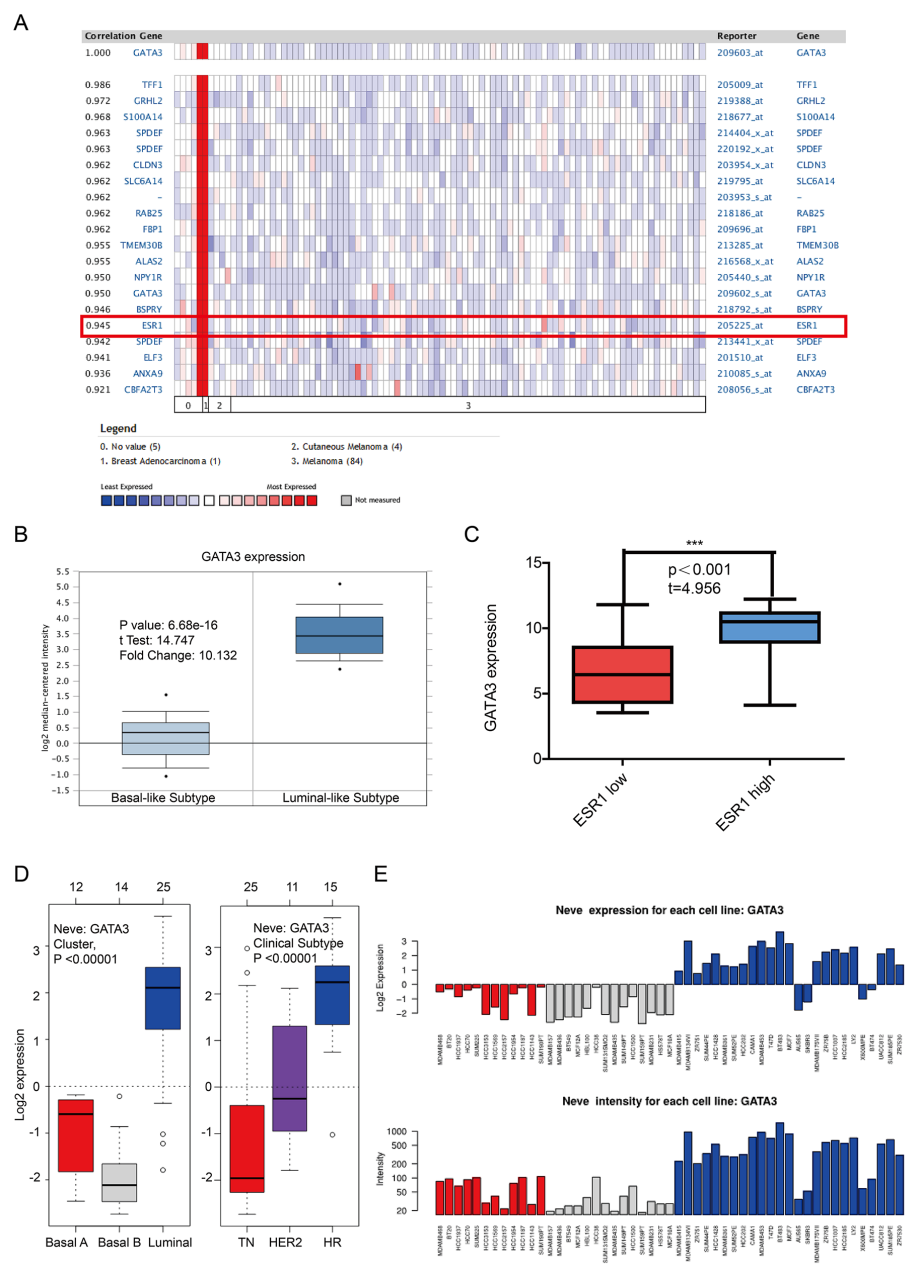
The correlation analysis of GATA3 in different molecular subtypes of breast cancer **(A)** In *ONCOMINE* analysis, the expression of GATA3 was significantly associated with ESR1 expression. (shown in red frame) **(B)** The expression of GATA3 in luminal-like was significantly higher than in basal-like subtypes of breast cancer. **(C)** In *CCLE* analysis, GATA3 over-expressed in the breast cancer cell lines with high level of ER expression, while under-expressed in those with low level or negative ER expression. **(D)** In *GOBO* analysis, the expression of GATA3 in luminal-like was significantly higher than in basal A or Basal B subtypes of breast cancer, and the Hormone Receptor (HR) subtype also express higher GATA3 than TN(Triple Negative) and HER2 clinical subtypes. **(E)** the expression of GATA3 in each cell lines.

**Figure 5:**
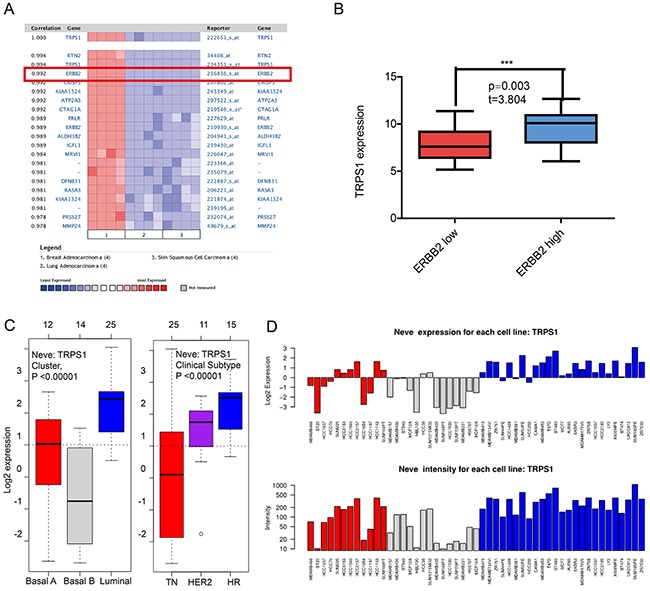
The correlation analysis of GATA3 in different molecular subtypes of breast cancer **(A)** In *ONCOMINE* analysis, TRPS1 expression was significantly correlated with ERBB2 expression. (shown in red frame) **(B)** In *CCLE* analysis, TRPS1 over-expressed in the breast cancer cell lines with high level of ERBB2 expression, while under-expressed in those with low level or negative ERBB2 expression. **(C)** In *GOBO* analysis, the expression of TRPS1 in different subtypes of breast cancer **(E)** the expression of TRPS1 in each cell lines.

Since GATA3 and TRPS1 were found to specifically high express in breast cancer among all GATA members, we next performed further exploration on the potential roles of GATA3 and TRPS1 in BC, in connection with other featured biomarkers according to molecular subtypes of breast cancer. In *ONCOMINE* co-expression analysis, it was found that GATA3 expression was significantly correlated with ESR1 (r=0.945) (Figure 4A), while TRPS1 expression was significantly correlated with ERBB2 (r=0.992) (Figure 5A). Dataset from Farmer's study [24] showed that GATA3 was 10.132 fold elevated in Luminal-like BC samples as compared with Basal-like BC (*p=*6.68*10e-16) (Figure 4B).

Similar results were found in *CCLE* analysis, GATA3 over-expressed in the breast cancer cell lines with high level of ER expression, while under-expressed in those with low level or negative ESR1 expression (*p*<0.001) (Figure 4C). TRPS1 over-expressed in the breast cancer cell lines with high level of ERBB2 expression, while under-expressed in those with low level or negative ERBB2 expression (*p*=0.003) (Figure 5B). The mRNA expressions of GATA3, TRPS1, ESR1 and ERBB2 in BC cell lines were shown in Supplementary Table.

In *GOBO* analysis, the expression of GATA3 in luminal-like was significantly higher than basal-A or basal-B subtypes of breast cancer, and the hormone receptor (HR) sensitive subtype also expressed higher level of GATA3 than TN (triple-negative) and Her-2 clinical subtypes (Figure 4D and 4E). The expression of TRPS1 in luminal subtypes was significantly higher than in basal A or basal B subtypes of BC both in tissue samples and cell lines (Figure 5C and 5D).

### GATA3 predicts longer RFS in patients with ER+/Luminal subtype breast cancer

**Figure 6:**
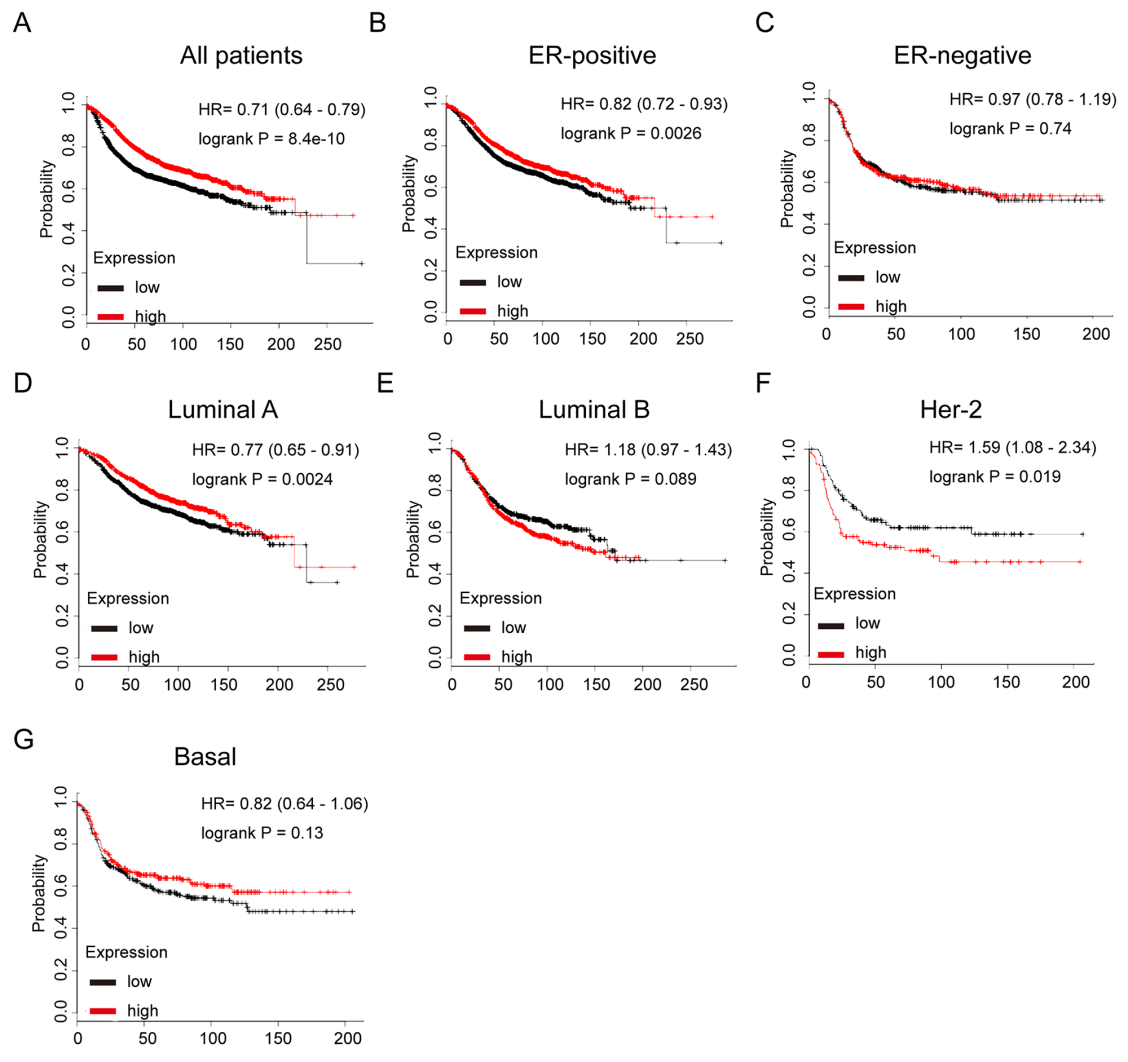
The prognostic values of GATA3 in breast cancer **(A)** High mRNA level of GATA3 was associated with longer RFS in all BC patients. **(B)** High mRNA level of GATA3 was associated with longer RFS both in ER+, but not ER- BC patients. **(C)** High mRNA level of GATA3 was associated with longer RFS in luminal A subtype BC patients, but not in luminal B subtype BC patients. **(D)** High mRNA level of GATA3 was not associated with RFS in either HER-2 positive or basal-like subtype BC patients.

We next assessed the prognostic effect of GATA3 in breast cancer. It was found that GATA3 mRNA high expression was correlated to longer RFS in all BC patients (HR=0.71, *p*=8.4e-10) (Figure 6A). In particular, sub-analysis revealed that GATA3 mRNA high expression was significantly associated with better RFS in both ER positive and luminal A subtype breast cancer (HR=0.82, *p*=0.0026 and (HR=0.77, *p*=0.0024, respectively), however, no significant difference was found in ER negative subtype, which suggested a pivotal prognostic value of GATA3 in ER positive or luminal subtype breast cancer, underlying the aberrant regulation of GATA3 in contributing to the tumorigenesis and development of hormone sensitive breast cancer (Figure 6B–6G).

### TRPS1 high mRNA expression is correlated to better RFS in patients with breast cancer, particularly in the subset who have received chemotherapy

**Figure 7:**
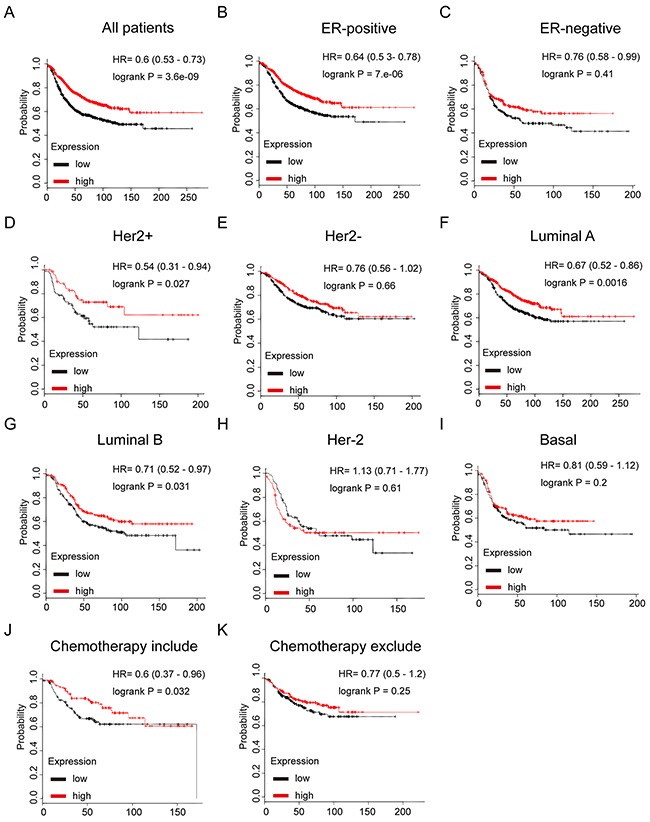
The prognostic values of mRNA level of TRPS1 in breast cancer patients **(A)** High mRNA level of TRPS1 was associated with longer RFS in all BC patients. **(B)** High mRNA level of TRPS1 was associated with longer RFS both in ER positive, but not in ER negative BC patients. **(C)** High mRNA level of TRPS1 was associated with longer RFS in luminal subtypes BC patients. **(D)** High mRNA level of TRPS1 is not associated with longer RFS in either luminal B or basal-like subtype BC patients. **(E)** High mRNA level of TRPS1 is associated with longer RFS in BC patients, who have received chemotherapy, while not associated with RFS in those haven't received chemotherapy.

In Figure 7, TRPS1 high mRNA expression was significantly associated with longer RFS in all BC patients (HR=0.6, *p*=3.6e-09) (Figure 7A). In particular, sub-analysis revealed that high mRNA expression of TRPS1 was significantly associated with better survival in ER positive (HR=0.64, *p*=7e-06), but not in ER negative BC (HR=0.76, *p*=0.41) (Figure 7B and 7C), furthermore, the results also demonstrated that TRPS1 high expression was significantly associated with longer RFS in luminal A (HR=0.67, *p*=0.0016), luminal B (HR=0.71, *p*=0.031), and Her-2 positive (HR=0.54, *p*=0.027), but not in Her-2 subtype (HR=1.13, *p*=0.61), Her-2 negative (HR=0.76, *p*=0.66) or basal-like subtypes BC (HR=0.81, *p*=0.2) (Figure 7D-7I).

Of noteworthy, the results demonstrated that TRPS1 high mRNA expression was significantly correlated to longer RFS in patients who have received chemotherapy (HR=0.6, *p*=0.032), indicating a potential role of TRPS1 in contribution to chemosensitivity in breast cancer (Figure 7J and 7K).

## DISCUSSION

Breast cancer (BC), mainly developing from the mammary epithelium, presents the highest prevalence and mortality among all malignancies in women, primarily attributing to resistance to chemoradiotherapy or targeted therapy, as well as distant metastasis [25–27]. It is challenging, but is going to be rewarding to illustrate the pathogenesis of BC, as well as to develop novel prognostic strategies and discover effective therapeutic approaches.

GATA family has been widely recognized as pivotal transcription factors in a wide range of human cancers [9, 28]. Our analysis suggested that, among all GATA members, GATA3 and TRPS1 were distinctively high-expressed in BC compared to normal controls, implying their unique roles in BC.

GATA3 is essential for multi-organ development and regulates tissue specific differentiation, especially for the development of mammary epithelium [29]. Asselin-Labat, M. L. et al. reported that, in a subtype of human breast carcinoma, GATA3 mutation abrogated the DNA-binding ability, with mechanistic investigation revealed that GATA3 negatively regulated the tumor-initiating capacity of mammary luminal progenitor cells and targets the putative tumor suppressor caspase-14 [15].

*ONCOMINE* co-expression analysis demonstrated that GATA3 was positively correlated with the expression of ESR1. The result was consistent with a meta-analysis of human cancer microarrays by Wilson, B. J. et al revealing that GATA3 was integral to the estrogen receptor alpha pathway [30].

Study by Wenzhe Si. et al reported that dysfunction of the reciprocal feedback loop between GATA3 and ZEB2-nucleated repression programs contributes to breast cancer metastasis, which indicated that expression of GATA3 in BC may associate with better RFS or DMFS (distant metastasis-free survival) [31]. Yoon et al also reported that higher levels of GATA3 predicted better survival in women with breast cancer [32]. Survival analysis of GATA3 in the present study demonstrated similar results that over-expression of GATA3 was associated with better survival in patients with breast cancer, particularly in patients with ER positive or luminal subtype breast cancer, suggesting the tumor-suppressive role of GATA3 in hormone sensitive breast cancer.

TRPS1 was reported to act as a crucial transcription factor for the development and differentiation of normal tissues, such as bone, hair follicles and kidney [33, 34]. Nonetheless, its role in cancer progression remains largely unknown. Study by Wu et al proposed that TRPS1 acted as a central hub in the control of cell cycle and proliferation during cancer development [35]. Our study found that TRPS1 was significantly high expressed both in BC samples and BC cell lines, and co-expressed with the expression of ERBB2, supporting the critical role of TRPS1 in BC initiation or progression.

Survival analysis indicated TRPS1 high expression was significantly associated with better RFS in all BC patients, hormone sensitive and Her-2 positive BC, but not in ER negative, Her-2 negative or basal-like subtypes, which implied that TRPS1 might act as a tumor suppressor in BC. A recent study by Huang et al. supported the notion and demonstrated that down-regulation of TRPS1 promoted EMT in a variety of cancer cells and was correlated with distant metastasis, tumor recurrence and poor survival rate in breast cancer patients [36]. Study by Stinson also suggested the suppressive role of TRPS1 in BC through inhibiting EMT in a directly repression of ZEB2 [37].

In our study, we first reported that high TRPS1 transcription predicted better survival in a subset of patients who have received chemotherapy, which was supported by a recent study showing that TRPS1 was associated with the multidrug resistance of osteosarcoma by regulating MDR1 gene expression [38]. Furthermore, it has been recognized that epithelial-to-mesenchymal transition (EMT) is an important mechanism in contribution to chemoresistance in breast cancer. Therefore, it is extrapolated that TRPS1 might be a favorable predictor of chemosensitivity in BC, with underlying mechanism possibly relates to suppression of EMT in breast cancer.

In conclusion, GATA3 and TRPS1 are distinctly high-expressed in breast cancer versus normal controls and predict better survival in patients with BC. GATA3 is positively associated with ESR1, while TRPS1 is correlated with ERBB2 and might act as a potential modulator of chemosensitivity in breast tumor. GATA3 and TRPS1 are distinctive biomarkers and essential prognostic factors in BC.

## MATERIALS AND METHODS

### ONCOMINE analysis

The mRNA levels of distinct GATA family members in different type of cancers were determined through analysis in *ONCOMINE* database (www.oncomine.org), which is a publicly accessible online cancer microarray database to facilitate discovery from genome-wide expression analyses.

In this study, students’*t*-test was used to generate a *p*-value for comparison between cancer specimens and normal control datasets. The fold change was defined as 2 and *p* value was set up at 0.01. Significant correlations can be found in an array of BC researches, as showed in typical figures.

### CCLE analysis

The mRNA levels of GATA3 and TRPS1 in a series of cancers were analyzed by *CCLE* database (https://portals.broadinstitute.org/ccle/home), which is an online encyclopedia of a compilation of gene expression, chromosomal copy number and massively parallel sequencing data from 947 human cancer cell lines, to facilitate the identification of genetic, lineage, and predictors of drug sensitivity.

### GOBO analysis

The transcription levels of GATA3 and TRPS1, as well as their co-expression genes were analyzed by uploading corresponding affymetrix probes to *GOBO* database (http://co.bmc.lu.se/gobo/gsa.pl). *GOBO* is a user-friendly online tool that allows rapid assessment of gene expression levels, identification of co-expressed genes and association with outcome for single genes, gene sets or gene signatures in a breast cancer data set.

The tumor data set consists of samples with the following characteristics: all tumors (n=1881), ER+ tumors (n=1225), ER- tumors (n=95), untreated tumors (n=927), tamoxifen treated tumors (n=326).

### The Kaplan-Meier plotter survival analysis

Prognostic values of featured GATA members that found specifically high expressed in BC samples were further assessed by displaying the relapse-free survival (RFS) using the Kaplan-Meier plotter (http://kmplot.com/analysis/) [39]. Kaplan–Meier survival curve, log-rank P value and HR with 95 % confidence intervals were calculated and plotted in R using Bio-conductor packages.

## SUPPLEMENTARY MATERIALS FIGURES AND TABLES




