# Supplementary material for: GATA3 and TRPS1 are distinct biomarkers and prognostic factors in breast cancer: database mining for GATA family members in malignancies
Source: Oncotarget. 2017 Mar 13;8(21):34750–61. doi: 10.18632/oncotarget.16160 (PMC5471008; doi:10.18632/oncotarget.16160)
Supplement: Supplementary file 1 [file oncotarget-08-34750-s001.pdf]

GATA3 and TRPS1 are distinct biomarkers and prognostic factors in breast cancer: database mining for GATA family members in malignancies

SUPPLEMENTARY FIGURES AND TABLE

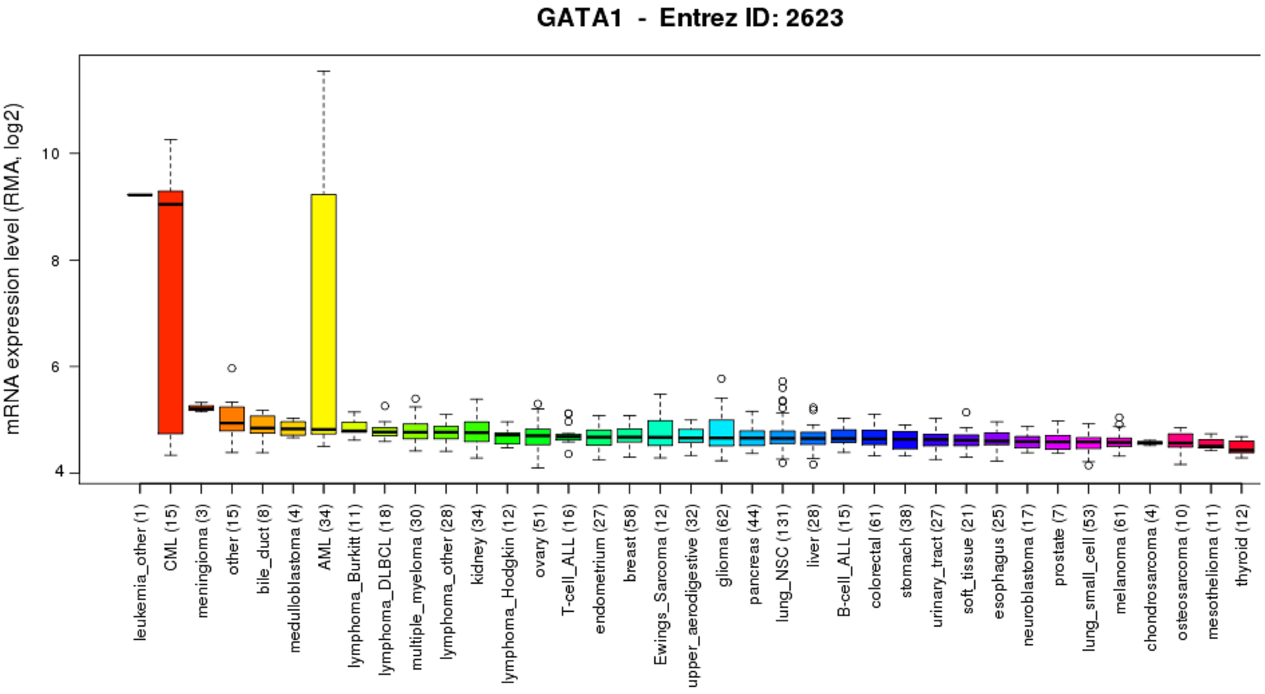

Supplementary Figure 1: The mRNA expression level of GATA1 (Entrz ID: 2623) in a variety of cancer cell line from CCLE analysis.

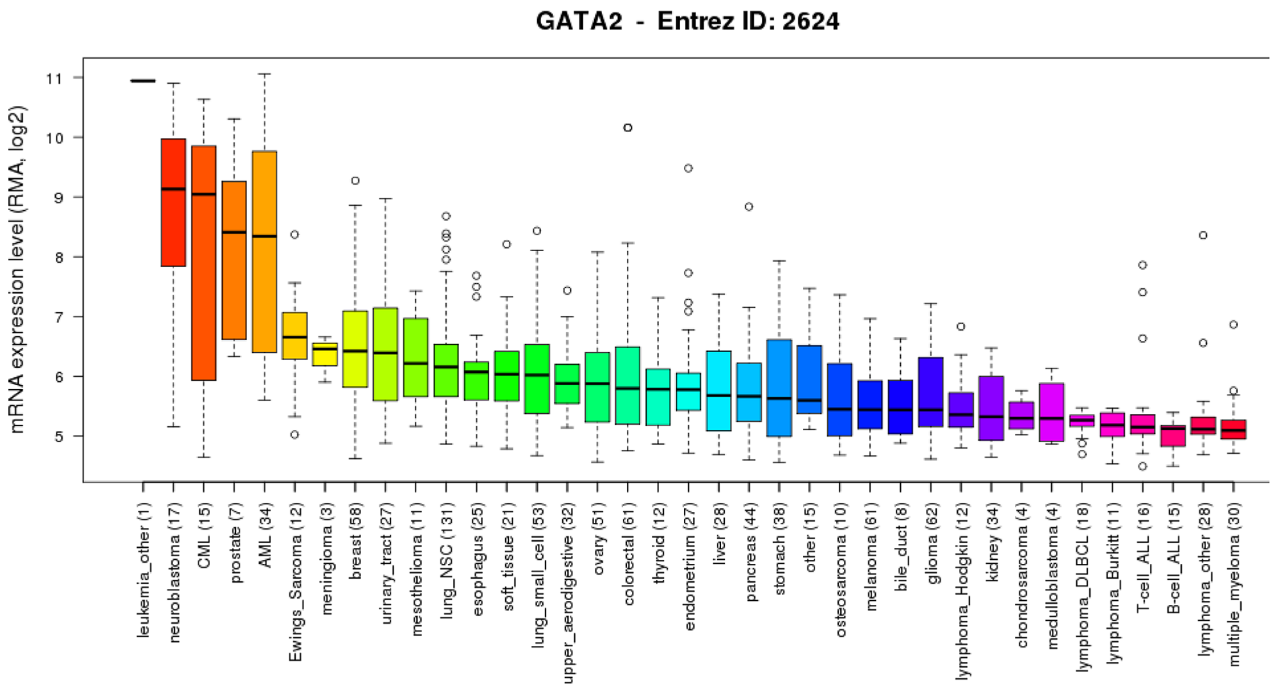

Supplementary Figure 2: The mRNA expression level of GATA2 (Entrz ID: 2624) in a variety of cancer cell line from CCLE analysis.

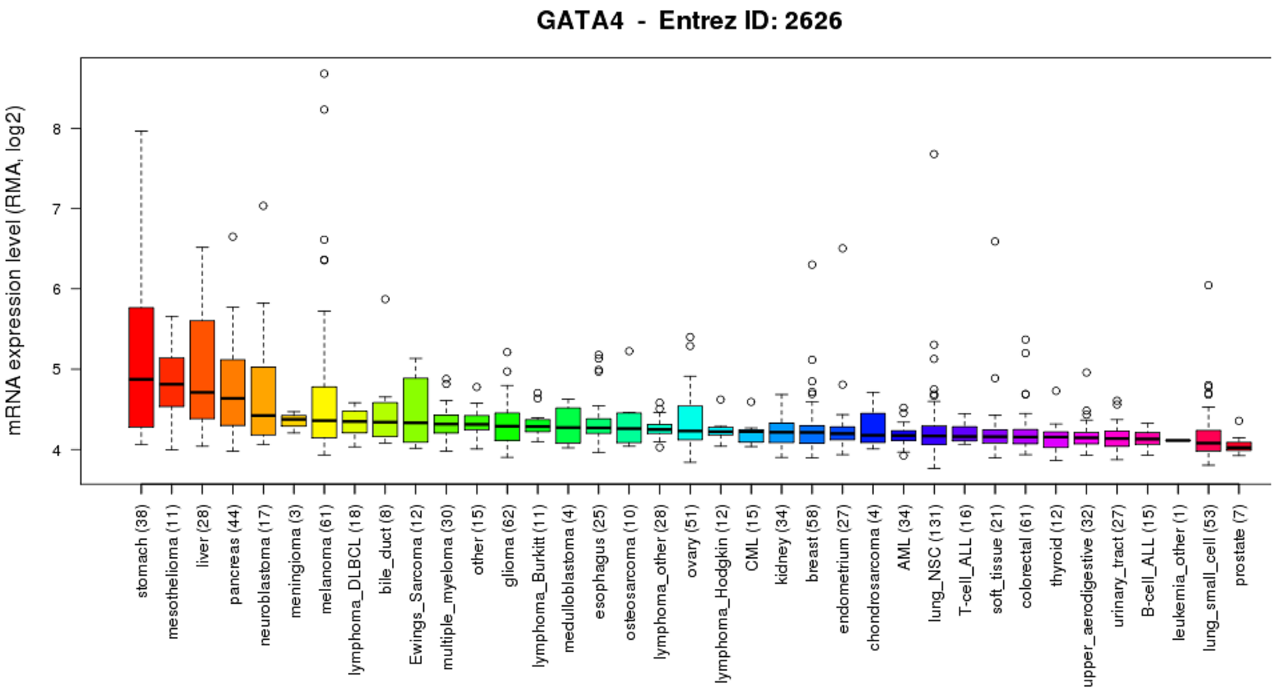

Supplementary Figure 3: The mRNA expression level of GATA4 (Entrz ID: 2626) in a variety of cancer cell line from CCLE analysis.

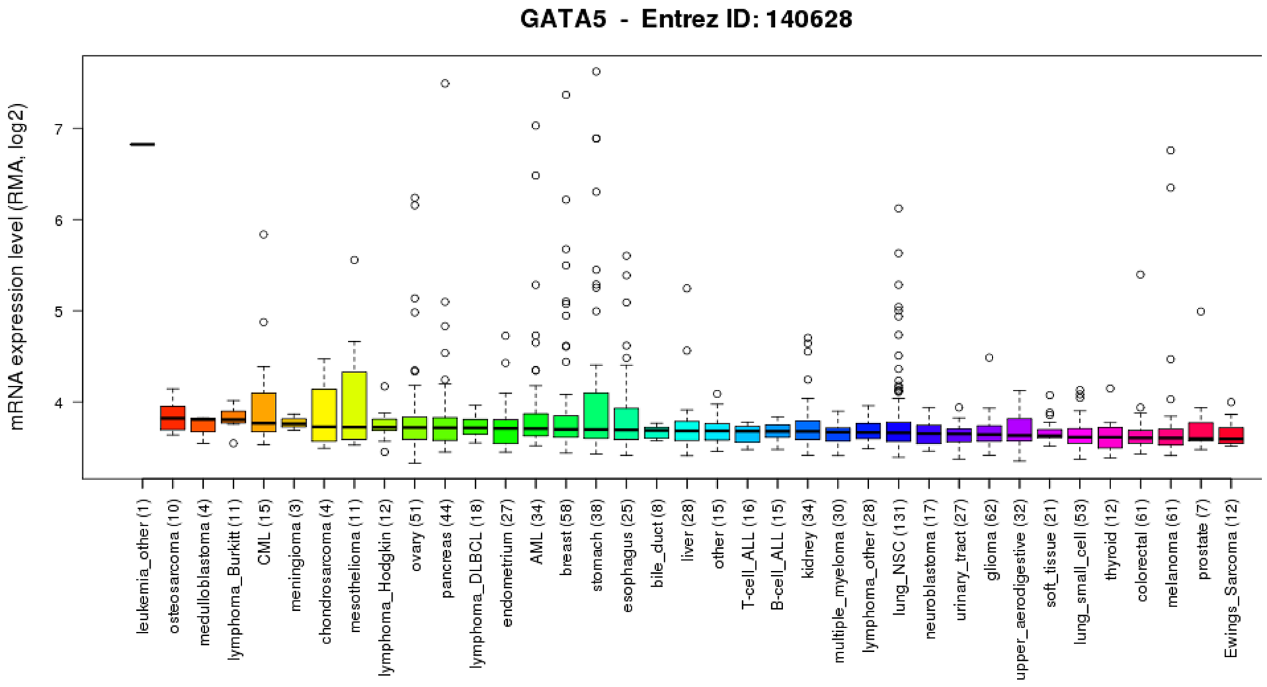

Supplementary Figure 4: The mRNA expression level of GATA5 (Entrz ID: 140628) in a variety of cancer cell line from CCLE analysis.

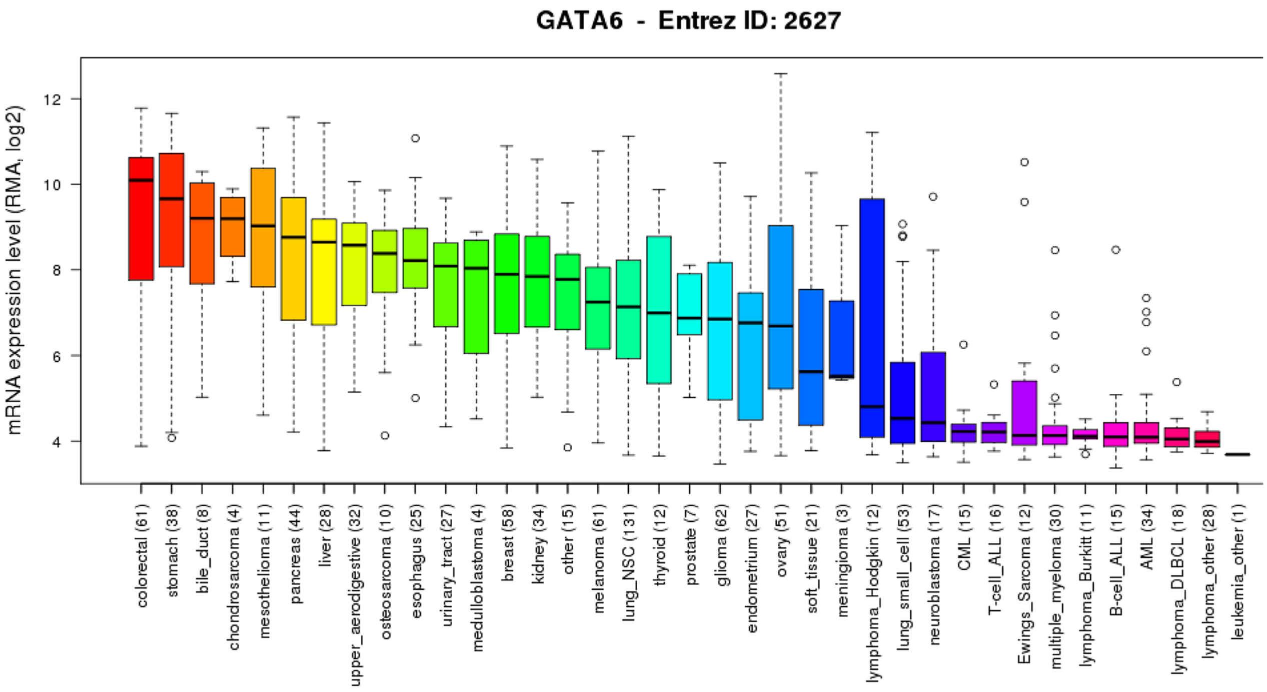

Supplementary Figure 5: The mRNA expression level of GATA6 (Entrz ID: 2627) in a variety of cancer cell line from CCLE analysis.

Supplementary Table 1: ER, ERBB2 and TRPS1 expressions in breast cancer cells.

See Supplementary File 1
