# Supplementary material for: GATA3 and TRPS1 are distinct biomarkers and prognostic factors in breast cancer: database mining for GATA family members in malignancies
Source: Oncotarget. 2017 Mar 13;8(21):34750–61. doi: 10.18632/oncotarget.16160 (PMC5471008; doi:10.18632/oncotarget.16160)
Supplement: Supplementary file 2 [file oncotarget-08-34750-s002.docx]

Supplementary Table: ER, ERBB2 and TRPS1 expressions in breast cancer cells

| cell line | ER | ERBB2 | GATA3 | TRPS1 |
| --- | --- | --- | --- | --- |
| MDAMB436_BREAST | 4.219699 | 6.235757 | 3.687624 | 8.217614 |
| HCC1395_BREAST | 4.253046 | 6.284023 | 4.006868 | 9.228217 |
| DU4475_BREAST | 4.516492 | 6.319157 | 4.581056 | 5.541925 |
| HMC18_BREAST | 4.670506 | 6.410262 | 4.116357 | 6.336863 |
| MDAMB468_BREAST | 4.555383 | 6.49737 | 8.092275 | 7.526193 |
| MDAMB134VI_BREAST | 7.614134 | 6.557435 | 10.75044 | 10.97934 |
| CAL120_BREAST | 4.538698 | 6.565585 | 10.18198 | 7.885957 |
| MDAMB231_BREAST | 4.449281 | 6.576142 | 6.302155 | 5.163587 |
| MDAMB157_BREAST | 4.530189 | 6.590551 | 3.539329 | 7.158966 |
| BT549_BREAST | 4.484761 | 6.635027 | 6.726012 | 8.703043 |
| HCC1143_BREAST | 4.814520 | 6.733552 | 7.196472 | 9.127327 |
| HS742T_BREAST | 4.611008 | 6.784908 | 3.860279 | 7.108999 |
| HS578T_BREAST | 4.274235 | 6.988897 | 5.329378 | 8.190734 |
| HCC1428_BREAST | 7.701086 | 6.994109 | 10.67714 | 11.06498 |
| HS606T_BREAST | 4.579861 | 7.000138 | 5.693747 | 6.813402 |
| HS343T_BREAST | 4.412126 | 7.004179 | 3.859108 | 6.492141 |
| HCC1599_BREAST | 4.805743 | 7.032977 | 4.131345 | 11.36612 |
| HS739T_BREAST | 4.658930 | 7.052768 | 5.543132 | 6.700677 |
| HCC1937_BREAST | 4.605427 | 7.147136 | 7.845543 | 7.610294 |
| HS274T_BREAST | 4.519405 | 7.215714 | 3.739647 | 6.373248 |
| HCC1500_BREAST | 7.228707 | 7.227412 | 11.2281 | 10.62468 |
| HS281T_BREAST | 4.556624 | 7.307357 | 5.176398 | 6.051501 |
| HCC1806_BREAST | 4.747687 | 7.343868 | 9.533081 | 5.511443 |
| MDAMB415_BREAST | 4.951737 | 7.415398 | 9.841284 | 10.38418 |
| HDQP1_BREAST | 4.546257 | 7.480261 | 9.479961 | 7.814634 |
| HCC70_BREAST | 4.583474 | 7.495977 | 9.002701 | 8.38746 |
| CAL51_BREAST | 4.355716 | 7.532843 | 4.441397 | 7.425822 |
| HMEL_BREAST | 4.951026 | 7.703873 | 5.950724 | 6.137638 |
| KPL1_BREAST | 5.857314 | 7.751493 | 12.04819 | 10.32908 |
| CAMA1_BREAST | 5.419593 | 7.899876 | 11.45469 | 10.84721 |
| CAL851_BREAST | 4.995962 | 7.90305 | 8.605885 | 6.122294 |
| MCF7_BREAST | 6.654008 | 7.905434 | 12.09107 | 10.05702 |
| BT20_BREAST | 4.614178 | 8.028439 | 9.021354 | 6.053737 |
| HCC1187_BREAST | 4.614561 | 8.070735 | 9.382199 | 7.710618 |
| T47D_BREAST | 6.785598 | 8.22675 | 11.58021 | 11.52854 |
| ZR751_BREAST | 5.905071 | 8.559273 | 10.14567 | 10.90483 |
| EVSAT_BREAST | 4.524186 | 8.631449 | 4.051028 | 9.736676 |
| HCC2157_BREAST | 4.721386 | 8.769267 | 4.416608 | 10.93573 |
| HCC38_BREAST | 4.314995 | 8.830835 | 7.746979 | 10.65195 |
| EFM19_BREAST | 7.027171 | 8.978568 | 10.9386 | 12.05513 |
| YMB1_BREAST | 4.801902 | 9.274765 | 10.39589 | 10.92485 |
| CAL148_BREAST | 4.368175 | 9.506454 | 7.433143 | 10.68574 |
| BT483_BREAST | 6.520489 | 9.580415 | 12.22645 | 12.67325 |
| MDAMB175VII_BREAST | 5.062837 | 9.65147 | 10.7867 | 10.40064 |
| MDAMB453_BREAST | 4.286688 | 10.05321 | 11.80195 | 11.15443 |
| JIMT1_BREAST | 4.540654 | 10.1783 | 9.65162 | 6.141853 |
| MDAMB361_BREAST | 5.564455 | 10.95687 | 9.304981 | 8.919209 |
| UACC812_BREAST | 5.517158 | 11.66212 | 11.59208 | 10.88551 |
| BT474_BREAST | 5.873165 | 11.78343 | 10.16078 | 10.15371 |
| SKBR3_BREAST | 4.386262 | 12.02384 | 7.510342 | 10.73933 |
| HCC1569_BREAST | 4.256883 | 12.14313 | 6.604398 | 10.94079 |
| HCC2218_BREAST | 4.819599 | 12.3154 | 10.51178 | 9.773872 |
| HCC1954_BREAST | 4.566255 | 12.63394 | 8.389632 | 7.22668 |
| HCC202_BREAST | 4.760342 | 12.72472 | 11.10348 | 10.13684 |
| ZR7530_BREAST | 5.642176 | 12.93231 | 10.75132 | 11.62906 |
| AU565_BREAST | 4.543484 | 12.9906 | 5.858272 | 9.487872 |
| EFM192A_BREAST | 5.316250 | 13.08469 | 9.403668 | 11.28502 |
| HCC1419_BREAST | 5.514727 | 13.16281 | 10.96785 | 11.43214 |
| UACC893_BREAST | 4.950250 | 13.47606 | 7.704794 | 8.41813 |
